# LIN28B Enhanced STAT3 Signaling Regulates Inflammatory Response and Chemotherapeutic Resistance in Cholangiocytes

**DOI:** 10.31557/APJCP.2021.22.11.3671

**Published:** 2021-11

**Authors:** Nattapong Puthdee, Supaporn Khramchantuk, Pattarin Nuwongsri

**Affiliations:** 1 *Department of Biochemistry, Faculty of Medicine, Chulalongkorn University, Bangkok, Thailand. *; 2 *Excellence Center for Stem Cell and Cell Therapy, King Chulalongkorn Memorial Hospital, the Thai Red Cross Society, Bangkok, Thailand. *; 3 *Graduate School, Faculty of Medicine, Chulalongkorn University, Bangkok, Thailand. *

**Keywords:** RNA-binding protein, tumor-initiation, inflammation, drugs resistance, bile duct cancer

## Abstract

**Background:**

LIN28B is functionally driving malignant transformation and relevance to the worse disease outcomes by promoting cancer aggressiveness. However, a typical role of LIN28B in cholangiocarcinoma (CCA) is primarily unknown. In this study, the tumorigenic potential of LIN28B in the cholangiocyte context was investigated.

**Methods:**

Stable LIN28B expression in MMNK-1 cells was generated by infecting with retrovirus-containing LIN28B gene. LIN28B-overexpressing cells were further validated the amount of released cytokines by using human cytokine arrays. After treatment of chemo-drugs, cell viability was subsequently measured using MTT assay. Aldehyde dehydrogenase (ALDH) activity was determined using ALDEFLUOR assay Kit and analyzed by flow cytometry. The mRNA and protein expression levels were respectively assayed by RT-qPCR and western blot.

**Results:**

Cytokine release results showed that numerous inflammatory cytokines-chemokines related to cancer initiation and development, such as IL-8, IL-6, VEGF, MCP1, TNF-α were significantly increased in LIN28B-overexpressing MMNK-1 cells. Drug sensitivity test showed that LIN28B-overexpressing MMNK-1 treated cells had a high percentage of cell viability compared to MMNK-1-control treated cells. Activity and expression level of a cancer stem cell marker, ALDH was significantly elevated in LIN28B-overexpressing MMNK-1 cells. Moreover, the activation of an oncogenic signaling pathway, signal transducer and activator of transcription 3 (STAT3) was enhanced in LIN28B-overexpressing MMNK-1 cells. Whereas, growth capacity of LIN28B-overexpressing MMNK-1 cells was found to be reduced in STAT3 inhibition.

**Conclusion:**

LIN28B can regulate the inflammatory response and resistance to chemotherapy of cholangiocytes through modulation of STAT3 signaling pathway.A recent study suggests that activated cholangiocytes can be induced by regulation of LIN28B/STAT3 pathway and this may partially contribute to the initiating CCA. Here, LIN28B and its downstream signaling could be considered as an attractive therapeutic target in patients with CCA.

## Introduction

Cholangiocarcinoma (CCA) is recognized as a devastating cancer with limited therapeutic options and poor prognosis. Due to lack of effective early diagnostic marker, patients were usually presented in the advanced stage of the disease (Blechacz et al., 2011; Bartella and Dufour, 2015).Thus, an extensively validated biomarker for early disease detection would be clinically beneficial. The knowledge of molecular mechanisms underlying early cellular transformation of CCA is required. Impaired inflammation is well known as an impact factor that plays a critical role in cholangiocarcinogenesis by causing genetic aberrationand activating several oncogenic signaling pathways (Andersen and Thorgeirsson, 2014). According to such knowledge, several potential therapeutic targets for CCA were suggested.However, the dominating molecular signaling which plays an especially early role in the transformation process is largely unclear.

LIN28B is an RNA-binding protein that plays pivotal roles in various cellular functions such as cell differentiation, metabolism, inflammation, and tumorigenesis (Viswanathan et al., 2009; Shyh-Chang and Daley, 2013). LIN28B regulates its targets through modulating mRNA stability and/ or enhancing their translation via suppression of let-7 miRNAs biogenesis (Newman et al., 2008; Viswanathan et al., 2008). In the presence of LIN28B, numerous mRNAs would not be degraded, including oncogenes such as cMyc, RAS, HMGA2, IMPs (Wang et al., 2016; Balzeau et al., 2017). An overexpression of LIN28B was found in various advanced human malignancies such as breast cancer, colon cancer, lung cancer, ovarian cancer and hepatocellular carcinoma and its expression was correlated with poor clinical outcomes of diseases (Zhang et al., 2019). Recently, c-Myc and LIN28B were reported to be upregulated in early steps of cholestasis accelerated CCA progression(Yang et al., 2011). Although this evidence indicates that LIN28B can partly contribute to CCA development,but the molecular mechanism of LIN28B in carcinogenesis is still unclear.

In this study, we hypothesized that LIN28B would play a role in the tumorigenesis of CCA. Therefore, we established stable LIN28B expression in cholangiocytes, MMNK-1 cells that closely mimic LIN28B-activating cells in disease progression and investigated the tumorigenic effect of LIN28B on normal cholangiocytes. 

## Materials and Methods

*Cell lines and cell culture*

Immortalized cholangiocyte MMNK-1 cells and bile duct cancer cells, HuCCT1 were obtained from JCRB cell bank and HEK293gp cell line was obtained from Stem Cell and Cell Therapy Research Center, Chulalongkorn University, Thailand. MMNK-1 and HEK293gp cells were maintained in Dulbecco’s Modified Eagle’s Medium (DMEM) (HyClone, USA) and HuCCT1 cells were cultured in RPMI1640 medium (Hyclone, USA). The culture medium supplemented with 10% fetal bovine serum, 1% Gluta Max and 1% Antibiotic-Antimycotic reagents (Gibco, USA). Cells were maintained in a humidified incubator at 37ºC with 5% CO_2_ atmosphere. 

*Viral production and establishment of stable gene-expressing cells*

The plasmids pBABE-hLIN28B and pBABE-control were gifted from George Daley. HEK293gp cells were transfected with the transfection reagents composing of plasmid encoding LIN28B or control, VSVG and X-tremeGENE HP DNA transfection reagent (Roche, Germany). After 72 h of transfection, the medium-containing viruses were collected and filtrated through 0.45 micron sterile filter. Prepared MMNK-1 cells and HuCCT1 were infected the virus by incubating with 1X polybrene (Sigma-Aldrich, USA) and viral containing LIN28B or empty vector control. The transfected cells were selected in medium containing 1 mg/ml of puromycin (Gibco, China) and cells were continuously maintained and cultured in medium supplemented with 1 mg/ml of puromycin.

*RNA extraction and RT-qPCR*

Total RNA was extracted using TRIzol reagent (Ambion, USA) following the manufacturer’s protocol. Briefly, the confluence cells were washed twice with cold PBS before adding 1 ml of the TRIzol reagents. Lysed cells was collected and then centrifuged at 12,000 rpm, 4ºC for 10 minutes. The clear supernatant was collected and mixing thoroughly in isopropanol, followed by chilling for overnight at -20ºC to precipitate the RNA. After centrifugation, RNA pellet was washed twice by adding 75 % ethanol. A total 1 ug of RNA was conducted to the synthesis of cDNA by using revertAid H Minus First Strand cDNA Synthesis Kit (Thermo Scientific, USA). cDNA sample was subjected for determination of mRNA expression by using SYBR Green PCR master mix (Thermo Scientific, Lithuania) and analyzed by real-time PCR (Applied Biosystem, USA). The mRNA expression levels were normalized to internal control, GAPDH. The relative gene expression or fold change was represented as 2^-∆∆CT^ by comparing to control sample. The primers sequences used in this study are following: for ALDH1A2; 5’-GCACGTCTGTCCCTCTCTGC-3’ (forward), 5’-TTGTGCAGTGACCTGCCTGG-3’ (reverse), for LIN28B; 5’-CATCTCCATGATAAACCGAGAGG-3’ (forward), 5’-GTTACCCGTATTGACTCAAGGC-3’ (reverse), for IL-6; 5’-CACACAGACAGCCACTCACCTC-3’ (forward), 5’-TCTGCCAGTGCCTCTTTGCTGC-3’ (reverse), for CDC25A; 5’-GAGATCGCCTGGGTAATGAA-3’ (forward), 5’-TGCGGAACTTCTTCAGGTCT-3’ (reverse), for CDK6; 5’-AGAGACAGGAGTGGCCTTGA-3’ (forward), 5’-TGAAAGCAAGCAAACAGGTG-3’ (reverse), for MCL-1; 5’-TTTGGCTACGGAGAAGGAGG-3’ (forward), 5’-ATAATCTCCAGCGACTGCCG-3’ (reverse). for GAPDH; 5’-CTGGGCTACACTGAGCACC-3’ (forward), 5’-AAGTGGTCGTTGAGGGCAATG-3’ (reverse). 

*Western blot*

Whole protein lysate was extracted using RIPA lysis buffer (Cell Signaling Technology, USA) mixing 1X Protease inhibitor cocktail (Thermo Scientific, USA).Cells lysate was centrifuged at 12,000 rpm for 10 minutes at 4ºC, and then the supernatant was collected. Protein concentration was measured using BCA Protein Assay by following the assay kit’s protocol (Thermo Scientific, USA). Protein samples (1 ug/ lane) were prepared and processed by using Simple Western assays (Protein Simple, USA), according to the manufacturer’s procedure and data was analyzed by using Compass for SW version 3.1.7 program. The following antibodies were used in this study:Stat3 (D3Z2G) Rabbit mAb, Phospho-Stat3 (Tyr705) Rabbit pAb, and β-actin (13E5) Rabbit mAb, Anti-rabbit IgG, HRP-linked antibody (7074S) were purchased from Cell Signaling Technology, USA. All antibodies were used in a dilution 1: 1,000. 

*Cell viability assay*

In brief, 5,000 cells/well were seeded into 96 well-plate. Next day, cells were treated with vary concentrations of chemotherapeutic drugs, 5-FU, Cisplatin, Gemcitabine, Etoposide (obtained from King Chulalongkorn Memorial Hospital) and STAT3 inhibitor; Cryptotanshinone (MedChem Express, USA). After indicated time, cells were incubated with 0.5 mg/ml of MTT (Sigma–Aldrich, USA) for 3 hours at 37 ºC. The formazan crystals were solubilized in DMSO and then absorbance at 570 nm. The percentage of cell viability was calculated by following the percentage of cell viability= (OD570 of treatment x 100)/mean OD570 of non-treated cells). 

*ALDH activity assay*

ALDH activity was performed by using ALDEFLUOR™ Kit (StemCell Technologies, USA). As following the manufacturer’s protocol, briefly, 1x10^6^ cells were suspended with 1 ml of ALDEFLUOR™ Assay Buffer. The activated ALDEFLUOR™ reagent was added and immediately transfers 0.5 ml of mixture to the tube containing DEAB solution, and then incubated for 45 minutes at 37ºC. ALDH fluorescence signal was detected using flow cytometry and analyzed by FlowJo^TM^ software v10.4 (BD Biosciences, USA).

*Cytokine array*

After cultured cells for 24 hours, the medium was collected and centrifuged at 1,000 xg, 15 minutes, 4ºC, and then the supernatant was collected. The amount of cytokines were measured using Bio-Plex Pro™ Human Cytokine 27-plex Assay (Bio-Rad, USA), according to the manufacturer’s instructions. The cytokine-standard and amount of cytokine in samples were analyzed using Bio-Plex 200systems (Bio-Rad, USA). 

*Immunofluorescence staining*

Cells-growing on a glass coverslip were then fixed by 4% paraformaldehyde in PBS for 10 minutes, and then incubated with permeabilization solution (0.3% Triton X-100 in PBS) for 10 minutes. After washed three time in PBS, cells were blocked non-specific binding by blocking buffer (5% normal goat serum in 0.3% Triton X-100 PBS) for 30 minutes. Cells were subsequently incubated with antibody (1:100) overnight at 4ºC. Cells were gently washed three times in 0.05% Tween20 PBS (PBST), and then incubated with Goat anti-rabbit IgG (H+L) secondary antibody FITC or Cy3 in a dilution 1:50 (Invitrogen, USA) for 1 hour. Lastly, cells were incubated with 1:100 of DAPI for 5 minutes (Invitrogen, USA). The fluorescently labeled cells were mounted by gold antifade mountant (Invitrogen, USA). The fluorescence signal was observed and captured the picture under the fluorescence microscope (ZEISS, Germany). 

*Trypan blue exclusion assay*

After incubation with chemotherapeutic agents for 72 h, cells were trypsinized and washed with PBS. Cells were mixed at 1:1 ratio with a 0.4% solution of trypan blue (Gibco, USA). The number of viable cells was measured using an EVE™ automatic cell counter (NanoEnTek Inc.). The percentage of cell viability was calculated, following the percentage of cell viability = (number of viable cells x 100)/ number of total cells. 

*Rhodamine 123 dye exclusion assay*

A total of 1x10^6^ suspend cells were incubated with 200 ng/ml of Rhodamine 123 dye (Sigma-Aldrich, USA) for 30 minutes on ice. Cells were then centrifuged at 1,200 rpm, three times for 5 minutes each in PBS to remove the excess of Rho123. Aliquots 500 μl of cell suspension (5x10^5 ^cells) and further analyze the retention of intracellular Rho123 using flow cytometry. Another part of cell suspension werethen centrifuged and further resuspended with warmed Rho123-free culture medium supplemented with 10% FBS, and subsequently incubated at 37ºC for 1 hour. After washing, the fluorescence Rho123 signals were then detected by using flow cytometry and data was analyzed by FlowJoTMsoftware v10.4 (BD Biosciences, USA).

*Statistical analysis*

Data were displayed as mean ± SD using GraphPad Prism software version 8.4.0 (USA). Student’s t-test was performed and P-value was considered statistically significant, indicating by *P≤0.05, **P≤0.01 and ***P≤0.001.

## Results

*Overexpression of LIN28B enhances the production of inflammatory cytokines in MMNK-1 cells*

**Figure 1:**
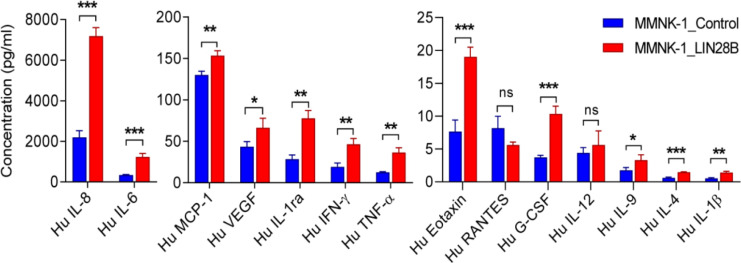
LIN28B Induces an Inflammatory Cytokines Production in MMNK-1 Cells. The concentrationsof cytokines release from LIN28B-overexpressing cells and control cells were measured by the human cytokine array. A bar graph represents mean±SD, triplicate wells. *P≤0.05, **P≤0.01, ***P≤0.001, ns indicates no significant

To explorethe tumorigenic potential of LIN28B in normal cholangiocytes, we then generated the stably overexpressing LIN28B in immortalized normal cholangiocyte, MMNK-1 cell lines. The successful LIN28B transfection was demonstrated by increasing LIN28B expression at the mRNA (Supplementary Figure 1a) and protein levels (Supplementary Figure 1b) in MMNK-1 cells. As such the knowledges, an inflammation is an important process that particularly occurs during CCA development and shown to be a critical role in the initiation and progression of CCA. Thus, we aim to investigate the effects of LIN28B on inflammatory response of cholangiocytes. As revealed by cytokine array, we found that the amount of release inflammatory cytokines such as IL-8, IL-6, MCP-1, VEGF, IFN-γ and TNF-α were significantly increased in LIN28B-overexpressing MMNK-1 cells (Figure 1). In the different contexts of LIN28B-expressing cells, we also found that the expression levels of IL-6 and IL-8 genes were increased in LIN28B-overexpressing HuCCT1 cells (Supplementary Figure 2a-b). The results indicate that LIN28B can induce inflammatory cytokine release, which this effect appear to be related with inflammatory response of cholangiocytes. 

*LIN28B increases ALDH activity and their resistance to chemotherapy*

**Figure 2:**
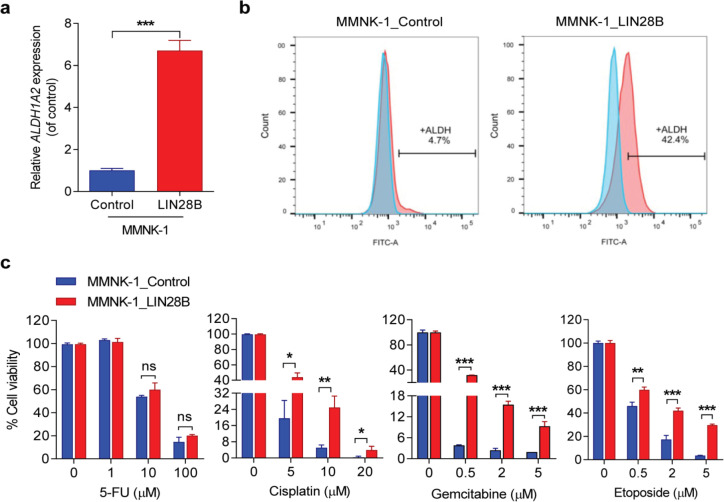
LIN28B Declines Chemosensitivity and Enhances ALDH Activity in MMNK-1 Cells. (a) The relative expression of ALDH1A2 gene was quantified by RT-qPCR and graph shown as fold change of gene expression levels in LIN28B-overexpressing MMNK-1 cells to control cells.(b) ALDH positive cells revealed by flow cytometry analysis. Light blue histogram indicates DEAB-negative ALDH control. (c) The percentage of cell viability of LIN28B-overexpressing MMNK-1 and control cells treated with vary concentrations of chemotherapeutic agents, including 5-FU, Cisplatin, Gemcitabine, and Etoposide for 72 hours were measured by MTT assay. Bar graphs represent mean±SD. n=3 .*P≤0.05, **P≤0.01, ***P≤0.001, ns indicates no significant

As we found LIN28B can induce the expression of several inflammatory cytokines. It is likely that stably LIN28B-overexpressing MMNK-1 cells exhibit the fundamental cancer properties. Thus, we further validated other characteristics of cancer cells. According to data of mRNA microarray analysis by our colleague revealed that the ALDH1A2 gene was significantly upregulated in the context of LIN28B expression (data not shown). As a result, we also validated in our study and found that ALDH1A2 gene was significantly elevated in LIN28B-overexpressing cells (Figure 2a, Supplementary Figure 2c). We subsequently determined the ALDH activity by using ALDEFLUOR assay, and found 4.7% ALDH-positive in MMNK-1 control cells whereas 42.4% ALDH-positive was found in LIN28B-overexpressing MMNK-1 cells (Figure 2b), and increasing of ALDH activity was also found in LIN28B-overexpressing HuCCT1 cells (Supplementary Figure 2d). According to role of the ALDH, its expression were specifically expressed and promoted anti-cancer drugs resistance in cancer stem cells (CSCs) (Clark and Palle, 2016). Hence, we then determined the inhibition effect of chemotherapeutic drugs on LIN28B-overexpressing cells. As revealed by MTT assay, we found that the percentage of cell viability of LIN28B-overexpressing MMNK-1 cells had significantly higher than control cells in Cisplatin, Gemcitabine, and Etoposide, but not 5-FU treatments (Figure 2c), whereas LIN28B-overexpressing HuCCT1 cells were shown to be resisted to Cisplatin and Etoposide (Supplementary Figure 2e). As demonstrated by trypan blue exclusion assay, confirmed that more viable cells were found in LIN28B-expressing cells (Supplementary Figure 3). These data suggest that LIN28B promotes chemotherapeutic resistance, which may partly be due to the responsibility of ALDH function. 

*LIN28B enhancesthe activation of STAT3 signaling*

**Figure 3:**
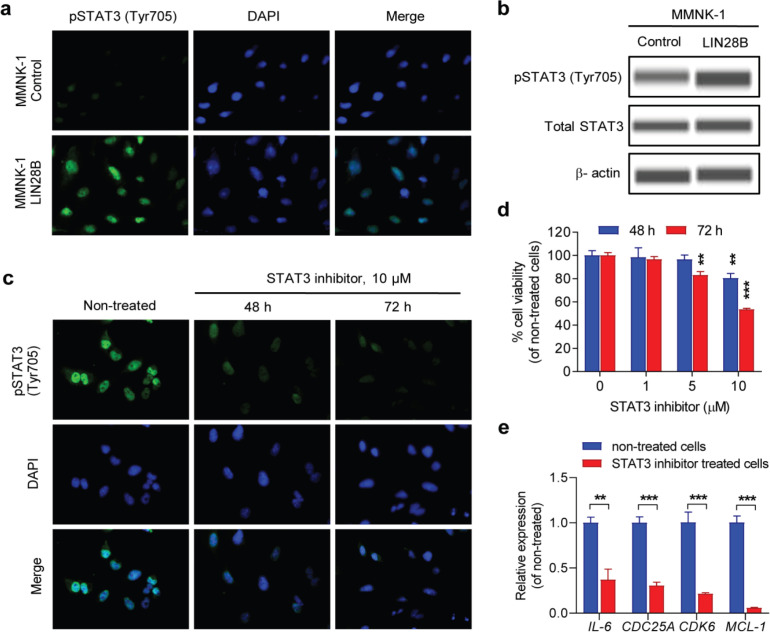
LIN28B Enhances Activated STAT3 Signaling. (a) Representative pictures of immunofluorescence staining showing the expression of phosphorylated STAT3 (Tyr705) in LIN28B-overexpressing MMNK-1 cells and MMNK-1 control cells. (b) Western blot indicating the expression of pSTAT3 (Tyr705) and total STAT3 proteins in LIN28B-overexpressing MMNK-1 cells and MMNK-1 control cells, β-actin is used for loading control. (c) Immunofluorescence staining representing the expression of pSTAT3 (Tyr705) in LIN28B-overexpressing MMNK-1 cells treated with 10 μM of STAT3 inihibitor for 48 h and 72 h . (d) MTT assay demonstrating the viable cells of LIN28B-overexpressing MMNK-1 cells treated with STAT3 inhibitor for 48 h and 72 h. (e) Real-time PCR showing the relative gene expression levels of IL-6, CDC25A, CDK6, and MCL-1 in LIN28B-overexpressing MMNK-1 cells treated with 10 μm of STAT3 inhibitor for 72 h. Bar charts show mean±SD, n=3. **P≤0.01, ***P≤0.001

We next investigated thepossible mechanisms involved in LIN28B promoting inflammation and survival of cholangiocytes. STAT3 signaling pathway is well known as a transcription factor that contributes to inflammation by regulating the expression of multiple pro-inflammatory cytokines (Yu et al., 2009). Importantly, aberrant STAT3 activation is associated with tumor promotion and progression of CCA (Sia et al., 2013). Thus, we then evaluated the STAT3 activation in LIN28B-overexpressing MMNK-1 cells and found that the phosphorylated STAT3 (Tyr705) was increased (Figure 3a-b). In contrast, the expression of pSTAT3 (Tyr705) was dramatically reduced in the STAT3 inhibitor treatment (Figure 3c). As a result, we expected that STAT3 may be a downstream signaling of LIN28B and it may be a possible target for against the effect of LIN28B. The result of MTT assay demonstrated that the potent STAT3 inhibitor significantly reduced cell viability of LIN28B-overexpressing MMNK-1 cells harboring constitutively active STAT3 signaling (Figure 3d). Moreover, downregulation of STAT3 targets such as inflammatory gene (IL-6), cell cycleregulatory genes (CDC25A and CDK6), and anti-apoptosis gene (MCL-1) were observed in STAT3 inhibitor treated cells (Figure 3e). Together, our findings indicate that LIN28B can enhance activated STAT3 signaling in cholangiocytes and this possible regulation may contribute to the tumorigenesis of CCA.

**Figure 4:**
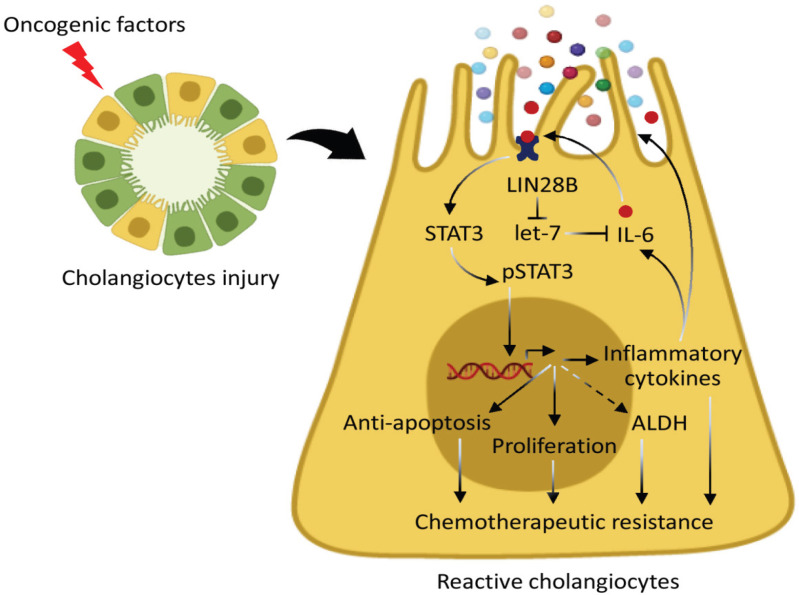
Propose a Potential Mechanism of LIN28B Induces Reactive Cholangiocytes. The diagram can be drawn by following the molecular cholangiopathy together with our findings. LIN28B would be activated follow by quiescence cholangiocyte exposed oncogenic inducing factors. The let-7 targets, IL-6 was subsequently upregulated in the context of LIN28B expression. The activated STAT3 signaling pathway resulted in transcription of IL-6 and it would turn to activate and create the inflammatory feedback loop, LIN28B/let-7/IL-6/STAT3. The effects of inflammatory cytokines on chemoresistance can be applied in LIN28B-overexpressing cholangiocytes by which their effects have been suggested to be involved in the promotion of chemoresistance by inducing pro-survival signals. Moreover, as a function of STAT3 could be applied in our context by which STAT3 primarily confers the chemotherapeutic resistance by inducing expression of several genes that play important functions in anti-apoptosis, cell proliferation, and as well as detoxifying ALDH enzyme

## Discussion

According to the critical role of LIN28B in tumorigenesis, it was reported to be a tumor-initiating factor and sufficient to induce cancer development in multiple contexts in vivo, including lymphoma (Beachy et al., 2012), neuroblastoma (Molenaar et al., 2012), and liver cancer (Nguyen et al., 2014). Nonetheless, the oncogenic role of LIN28B in CCA is largely unknown. Based on the pathogenesis of CCA, an inflammation process is typically associated with the pathogenesis of bile duct epithelial then allowing the accumulation of proto-oncogenes mutations and impairing of tumor suppressor genes, resulting in CCA formation (Zabron et al., 2013). We have revealed that LIN28B can induce the production of inflammatory cytokines, including IL-6, IL-8, MCP-1, VEGF, IFN-γ, and TNF-α in cholangiocytes. The elevated cytokines were shown to be tumor-promoting inflammatory cytokines and significant role in the carcinogenesis of CCA (Banales et al., 2020). According to the significant role of IL-6/ STAT3 pathway in tumorigenesis of CCA (Rizvi and Gores, 2014; Roy et al., 2019), implying that LIN28B may partially involve in CCA initiation by inducing an inflammatory loop IL-6/STAT3 in cholangiocytes. Moreover, our findings also agrees with a previous study by which LIN28B/let-7 axis can sustain an inflammatory positive feedback loop, NF-κB/LIN28B/let-7/IL-6/STAT3 in normal breast -transformed cells (Iliopoulos et al., 2009). Together, considering that LIN28B/IL-6/STAT3 regulation seems to be an inflammatory loop-driven CCA initiation and able to explain the function of LIN28B in an early step of cholestasis -accelerated CCA in vivo (Yang et al., 2011). 

Other elevated cytokines in LIN28B-expressing cholangiocytes are particularly produced and released by the cholangiocytes and their surrounding microenvironment during carcinogenesis of CCA (Al-Bahrani et al., 2013). Their autocrine/ paracrine effects are crucial for inflammatory response and cause bile duct epithelial cell injury (Raggi et al., 2015). TNF-α and IFN-γ can alter biliary barrier integrity and affect to cholangiocyte choleretic activity(Shinichiro et al., 2003). TNF-α, IL-4,and IL-6 can promote the epithelial-mesenchymal transition (EMT) in CCA cell lines by increasing the expression of EMT-related genes (Techasen et al., 2012a; Techasen et al., 2012b). IL-8 and MCP-1 can induce leukocytic infiltration into inflammatory site, and causing cholangiopathic injury (Syal et al., 2012). VEGF is subsequently released by injured cholangiocytes, and its autocrine and paracrine effects stimulate cholangiocyte proliferation (Mariotti et al., 2021). It seems that the releasing of many inflammatory cytokines of LIN28B-expressing cholangiocytes are likely exhibited as the activated cholangiocytes features, which are associated with biliary pathophysiology, including cholestasis, proliferation, survival, inflammation, development of biliary fibrogenesis, and eventually biliary carcinogenesis (Banales et al., 2019). Notably, these elevated cancer -promoting inflammatory cytokinesare regulated by STAT3 pathway (Yu et al., 2009), indicating that LIN28B possibly promotes malignant cholangiocytes transformation by inducing the expression of tumor -promoting inflammatory cytokines via STAT3 signaling pathway. Thus, this study provides an insight of molecular mechanism of LIN28B/ STAT3 into the tumor-initiation capacity of CCA.

Furthermore, we demonstrated that LIN28B-overexpressing cholangiocytes exhibit low sensitivity to Cisplatin, Etoposide, and Gemcitabine. LIN28B is likely promoted drug resistance by following themulti-factorials mechanistic of chemoresistance (MOCs) (Marin et al., 2018).The reduction of intracellular drug activity (MOC-2) is proposed by which LIN28B induces the detoxifying ALDH enzyme activity, which has been shown to reduce the effect of gemcitabine and drug resistance in CCA patients (Chen et al., 2016). The expression of ALDH and its activity is specifically found in CSCs sub-populations (Xu et al., 2015) and it has functionally contributed to anti-cancer drugs resistance of CSCs by reducing the cytotoxicity of various harmful aldehydes and alkylating agents (Clark and Palle; Toledo-Guzmán et al., 2019). Considering that LIN28B may potentially promote cancer stem-like properties, chemotherapeutic resistance in cholangiocytes by enhancing ALDH activity. However, the mechanism of LIN28B regulates ALDH expression is unknown. Previous studies have shown that co-expression of LIN28B and ALDH1 isoform was significantly found in cancer stem-like cells and tumor tissues (Chien et al., 2015; Wu et al., 2017). Moreover, the functional studies demonstrated that ALDH expression and its activity in ALDH-positive prostate cancer cells were significantly decreased when inhibited the activation of STAT3 (Han et al., 2014), as well as data shown in CCA cell lines (Beyreis et al., 2019). For that reason, ALDH is likely to be a downstream target of STAT3, hence, implying that LIN28B induces ALDH expression via modulating STAT3 signaling in our context. It will be of interest to next investigate the mechanisms of how LIN28B regulates ALDH in other contexts. 

Moreover, LIN28B may implicate in other MOC by which LIN28B induces cholangiocyte secretion of many cytokines associated with ductular reaction which are known to promote apoptosis inhibition (MOC-5a) and cholangiocyte proliferation (MOC-5b) (Sato et al., 2019), those cytokines also play an important role in regulating immune cell activity in the tumor microenvironment (MOC-6) (Pinto et al., 2018). On the other hand, the activity of drug efflux transporter (MOC1b) may not involve in LIN28B declined the inhibition effects of chemotherapeutic drugs in cholangiocytes (Supplementary Figure 4). We also noticed that the viability of cells treated 5-FU was not significantly changed, which is likely due to the normal cholangiocytes are already existed 5-FU metabolizing enzymes, NAD(P)H-quinone oxidoreductase 1 (NQO1)(Zeekpudsa et al., 2014) and rate-limiting enzyme, dihydropyrimidine dehydrogenase (DPD) (Arunsan et al., 2020).

Collectively, we demonstrate that LIN28B enhanced STAT3 signaling causes inflammatory cytokines production and declines chemotherapeutic drugs sensitivity while increasing cancer stem cell markers, ALDH activity. According to such knowledge of STAT3 regulation and our findings, we propose the potential role of LIN28B induces CCA malignant transformation, as shown in Figure 4. This finding provides the evidence that LIN28B/IL-6/STAT3 signaling cascade may be involved in carcinogenesis of CCA and also suggests LIN28B and its downstream signaling may be an interesting therapeutic strategy for patients with CCA.

## Author Contribution Statement

Puthdee N; experimental design, drafting original and revision of the manuscript, carrying out the experiments, and data analysis and interpretation. Khramchantuk S; carrying out the experiments and data interpretation. Nuwongsri P; citation of references and preparation of figures. All authors approved the final version of the manuscript. 

## Funding statement

This study was supported by Ratchadapiseksompotch Funding, Faculty of Medicine, Chulalongkorn University, No. RA(MF)05/59 and RA(MF)12/60. Puthdee. N is supported by Chulalongkorn University graduate scholarship to commemorate the 72nd anniversary of his majesty king bhumiboladulyadej. 

## Ethical approval

This study was ethically approved from Institutional Review Board of Research Affairs, Faculty of Medicine, Chulalongkorn University, (IRB. No 348/60). 

## Conflict of interest

The authors declare no potential conflicts of interest
